# Association of Transcranial Direct Current Stimulation and Neurofeedback With Declarative Memory and Cerebral Arterial Flow in University Students: Protocol for a Double-blind Randomized Controlled Study

**DOI:** 10.2196/36294

**Published:** 2022-08-26

**Authors:** Leandro H Grecco, Giuliano R Gonçalves, Bárbara Neiva Perri, Breno Alexander Bispo, Isabella Favilla Jorge Grandin, Paula Valentina Nunes Dias Gomes, André Alexandre Bocchi, Kennedy Martinez Oliveira, Diogo Correa Maldonado, Marcelo Cavenaghi Pereira da Silva

**Affiliations:** 1 Department of Morphology and Genetics Universidade Federal de São Paulo São Paulo Brazil; 2 Faculdade São Leopoldo Mandic Campinas Brazil; 3 Universidade Federal de São Paulo São Paulo Brazil; 4 Pontifícia Universidade Católica de Campinas Campinas Brazil; 5 Anatomy and Image Department Faculty of Medicine Universidade Federal de Minas Gerais Belo Horizonte Brazil

**Keywords:** transcranial direct current stimulation, neurofeedback stimulation, TDCS, memory, Doppler ultrasonography, arterial flow, brain stimulation, electrical stimulation, transcranial, neurofeedback, brain, neurology, cerebral, blood flow, cerebrum, declarative memory, double-blind, controlled trial, RCT, randomized controlled trial, university, college, student, postsecondary, graduate, undergraduate

## Abstract

**Background:**

The performance of a task depends on ongoing brain activity, which can be influenced by attention, excitement, or motivation. Scientific studies have confirmed that mindfulness leads to better performance, health, and well-being. However, these cognitive efficiency modulating factors are nonspecific, can be difficult to control, and are not suitable to specifically facilitate neural processing.

**Objective:**

The aim of this study is to evaluate the effects of transcranial direct current stimulation associated with neurofeedback on declarative memory and cerebral blood flow in university students.

**Methods:**

In this study, we will use transcranial direct current stimulation, a low-cost physical resource that is easy to apply, has few adverse effects, and is associated with a neurofeedback resource. This, in turn, has been shown to be a training program capable of improving working memory function.

**Results:**

Participants will be recruited between July 2022 and December 2022. This study is expected to conclude in July 2023.

**Conclusions:**

This study will provide preliminary results on the benefits of using the direct current neurostimulation and neurofeedback tools on the participants being analyzed.

**Trial Registration:**

Brazilian Clinical Trials Registry RBR-7zs8b5; https://ensaiosclinicos.gov.br/rg/RBR-7zs8b5

**International Registered Report Identifier (IRRID):**

PRR1-10.2196/36294

## Introduction

As is well known, learning and memory involve explicit procedures (eg, declaratives) and implicit processes (eg, procedures), and improving one or both can contribute to improving learning [1]. Working memory refers to the temporary storage and manipulation of information needed for complex tasks such as language comprehension, attention, learning, and reasoning. One of the main goals of educational efforts is to develop techniques for improving learning and promoting better retention [1,2].

Today computer games are being developed not only for gamer entertainment but also in the field of health and education to improve one or more of the cognitive abilities of users. These games can improve problem-solving skills [3,4]. Brain events can be recorded in a noninvasive and flexible way through the electroencephalogram (EEG), and EEG-based applications were initially developed to help people with disabilities to communicate with machines; later they were used in video games as controllers and more recently as neurofeedback games [5]. Neurofeedback is a noninvasive, drug-free form of brain training reported to help with a variety of conditions, including pain, fatigue, depression, anxiety, sleep disturbances, and cognitive decline [6]. We can also include the implementation of a game to test the progress of trained individuals with a more flexible and interesting approach. It is a simple game application that uses visualization techniques to increase enthusiasm without being aware of being tested. As the system makes use of a participant’s direct attention to control a game, it works on a feed-forward mechanism [7]. Neurofeedback offers the possibility of endogenously manipulating brain activity as an independent variable, making it a powerful neuroscientific tool [8]. Some areas of clinical research involve comparing or combining neurofeedback with other interventions such as pharmacotherapy, behavioral therapy, and neurostimulation (eg, transcranial direct current stimulation [TDCS]).

TDCS noninvasively induces plasticity-related changes in neural circuits in vivo and is experiencing increasing use as a potential tool to modulate brain function [9]. There is growing evidence that TDCS-related outcomes are likely to be influenced by an individual’s brain state at the time of stimulation, that is, the effects show a degree of “state dependence” [10]. Fregni et al [11] demonstrated that anodic TDCS in the left dorsolateral prefrontal area leads to an increase in working memory performance, that is, increasing the accuracy of a task when compared to simulated stimulation of the same area. This study corroborates literature data showing TDCS as a tool that can significantly impact some aspects of knowledge [12].

TDCS is characterized by the administration of a single-phase electrical current of low intensity (0.5-2 mA) applied to the scalp through surface electrodes. This stimulation induces lasting changes in cortical neuronal excitability, both in animals and in humans [9]. TDCS-induced cortical modulation is dependent on the polarity of the applied current, and the effects are obtained by the movement of electrons. The poles of the electrodes used are the anode and cathode, with the anode being the positive pole and the cathode the negative pole. During the application of TDCS, the electrical current generated by the electrodes penetrates the skull, reaching the cortex [13]. Although most of the current dissipates between tissues superficial to the cortex, a sufficient amount of current reaches the cortical structures, thus modifying the membrane potential of the cells located there. It has been observed that anodic current increases cortical excitability, favoring neuronal membrane depolarization, while the cathodic current has an inhibitory effect by hyperpolarizing the neuronal membrane. TDCS has been shown to be a useful tool in the treatment of neuropsychiatric diseases and physical rehabilitation processes, being a safe and inexpensive form of noninvasive brain stimulation [10,11,14].

Some studies covering healthy individuals support the hypothesis that TDCS improves performance involving working memories, compared to simulated stimulation (placebo), and these effects can last up to 30 minutes after the end of the session. Nitsche et al [15] proposed general exclusion criteria for TDCS in healthy individuals: individuals must be free from unstable medical conditions or any disease that may increase the risk of stimulation, for example, neurological diseases such as epilepsy or acute eczema under the electrodes. Additionally, they must not have metallic implants near the electrodes. Side effects such as itchiness, a burning sensation, or a headache are common but usually mild and with no long-term impact. Thus, TDCS compares favorably with other therapeutic approaches such as antidepressants or transcranial magnetic stimulation [15].

Noninvasive neuromodulation has emerged as an alternative to replace other forms of treatment such as pharmacological treatments, which although effective, have a high rate of side or adverse effects. Among the different types, neurofeedback and TDCS have shown to be promising approaches that can modify brain wave oscillation and can be used in the development of skills for the self-regulation of brain activity [16]. However, no data were found in the literature associating these two features. The aim of this trial is to investigate the effectiveness of TDCS on the dorsolateral prefrontal cortex (DLPFC) associated with neurofeedback evaluating behavioral and physiological parameters of healthy college students. We hypothesize that there will be a significant increase in declarative memory responses and increased cerebral blood flow using the proposed protocol.

## Methods

### Study Design

This is a prospective, parallel, double-blind, randomized controlled, and 2-armed clinical trial. This study will be single center and carried out on the premises of Faculdade São Leopoldo Mandic/Campinas, São Paulo, Brazil.

**Figure 1:**
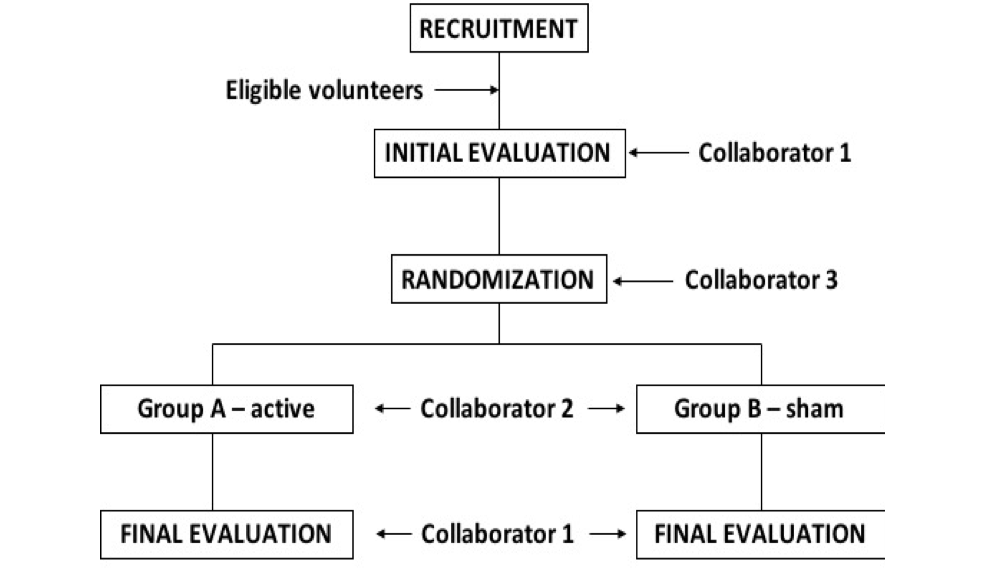
Flowchart of study design.

After an initial evaluation carried out by a research collaborator (collaborator 1), the participant will carry out the research protocol with another collaborator (collaborator 2) according to the draw performed by this collaborator. At the end of the protocol, the participant will again undergo an evaluation that will be performed by collaborator 1. Only collaborator 2 will know if the participant performed active stimulation or placebo. Therefore, this procedure will allow blinding of the participant and collaborator 1 (evaluator) to the stimulation conditions (Figure 1).

### Sample

The sample of 50 volunteers was calculated assuming a 40% difference in the proportion between the active TDCS and placebo groups in performance before and after the interventions. This calculation was performed using an online statistical program, considering an alpha of .05 and a power of 0.80, resulting in a sample of 25 individuals in each group (intervention and control). To approximate unexpected factors, we applied a 20% dropout rate, reaching a total sample size of 60 individuals.

### Inclusion Criteria

Eligible volunteers for the study must meet all the following items prior to randomization: medicine and dentistry undergraduates in their first to eighth semester, duly enrolled in the institution; aged between 18 and 30 years; and without distinction of sex or gender.

### Exclusion Criteria

Participants will be ineligible for the study if their medical history present psychiatric disorders, neurological trauma, epilepsy, seizures, or drug abuse in the last 6 months (including alcohol); if they are using medication that acts on the central nervous system by self-report or have any metallic implant in the head region; and if they are pregnant or planning to get pregnant in the next 2 months.

### Randomization and Blinding

Once eligibility and consent have been approved and completed, randomization will occur using the random list generated by an automated web-based randomization program. Participants will be randomly assigned into 1 of the following 2 groups:

1. Group A: TDCS active + neurofeedback
2. Group B: placebo TDCS + neurofeedback

Participants randomized to receive placebo TDCS will have the opportunity to enroll in an open-label portion of the study upon completion of their participation in the randomized portion of the study.

This process will be carried out by a member of the research team (collaborator 3) who is not involved in the study recruitment or development process.

### Ethical Approval

In accordance with the Declaration of Helsinki, this study strictly follows ethical principles in research involving human participants. All participants will be informed of the nature of the study and all procedures prior to registration. Following resolution 196/96 of the National Health Council (Brazil), only those who sign the free and informed consent form will be included.

This study was approved by the Ethics and Research Committee of Faculdade São Leopoldo Mandic/Campinas-SP under the opinion number 08051619.1.0000.5374. Furthermore, this study was submitted to the Brazilian Registry of Clinical Trials and approved under the RBR-7zs8b5 protocol (Universal Trial Number U1111-1254-0883).

### Assessments

Once eligibility and consent have been approved and completed, volunteers will undergo the Stroop test (based on the study by Campanholo et al [17]). This test consists of two tasks, one for reading and the other for naming the color. In both, the stimuli are color names printed in incongruous colors. The word-reading task gives an indication of reading fluency and serves to establish a benchmark for performance effectiveness relative to the color-naming task. The fact that there is an incongruity between the word name and the ink color causes an interference effect in the color naming. This interference is the Stroop color effect. Tests inspired by the Stroop effect are widely used in neuropsychology to measure individuals’ executive control and concentration [17].

After performing the Stroop test, the participant will undergo an assessment of cerebral blood flow by means of a transcranial Doppler ultrasound examination of the middle cerebral artery. The protocol developed by Rogge et al [18] will be carried out. Color-coded transcranial Doppler ultrasound will be performed in combined color and power mode with an ultrasound system equipped with a 2.5 mHz multifrequency probe transducer. Transtemporal insonation will be performed through the temporal bone window using axial and axial insonation plans and coronal with the participant in the right dorsal decubitus position. No eco-enhancer contrast will be used. Peak systolic and end diastolic pressure velocities will be measured in pulsed wave mode. Flow velocity measurements will be performed without angle correction. The examiner was free to optimize the images by changing the pulse gain and repetition frequencies [18].

### Interventions

#### Transcranial Direct Current Stimulation

**Figure 2:**
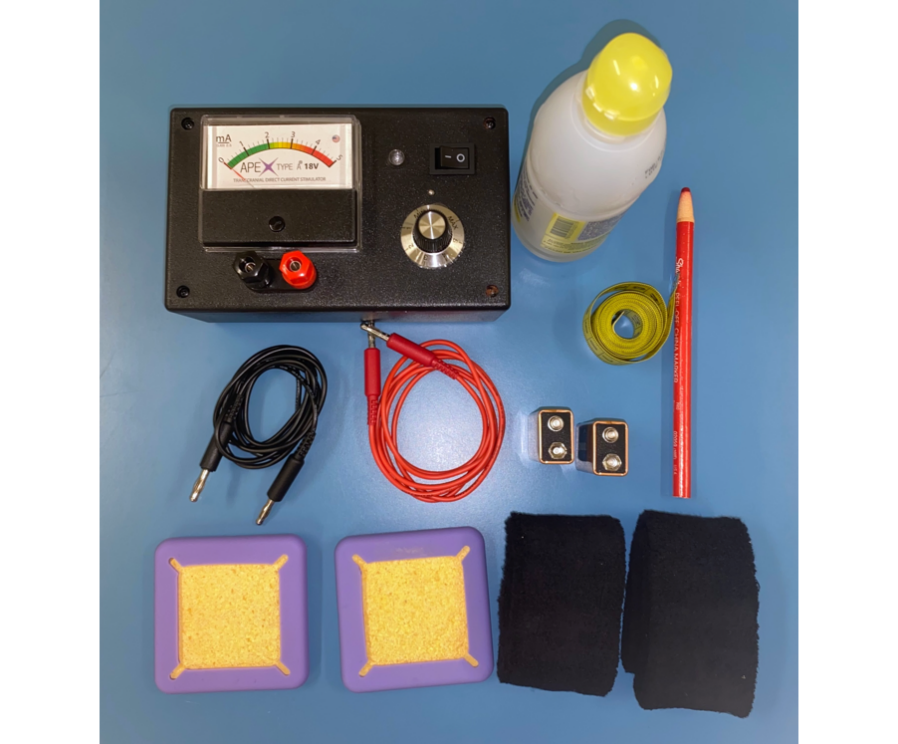
Transcranial direct current stimulation device.

A direct current will be applied by a pair of spongelike surface electrodes soaked in saline (35 cm^2^) and supplied by a specially developed constant current stimulator with a maximum output of 10 mA. To stimulate the DLPFC, the anode electrode will be placed over F3 according to the international 10-20 system for EEG electrode placement. The cathode will be placed over the contralateral supraorbital area (Figure 2).

For simulated stimulation, the electrodes will be placed in the same position; however, the stimulator will turn off after 20 seconds as described above. Therefore, the individuals felt the initial itching sensation at first, but they received no current for the remainder of the stimulation period. This procedure will allow the individuals to be blinded to the conditions of stimulation. A total of 10 stimulation sessions will be held for 2 consecutive weeks, with an interval of 2 days between the weeks. The duration of each session is 20 minutes. The applied intensity will be 2.0 mA [11,12,14].

Possible adverse effects are a tingling sensation and redness at the electrode site after stimulation. Headaches and burns may rarely occur. Possible side effects will be evaluated on a daily basis using a previously prepared questionnaire.

#### Neurofeedback

**Figure 3:**
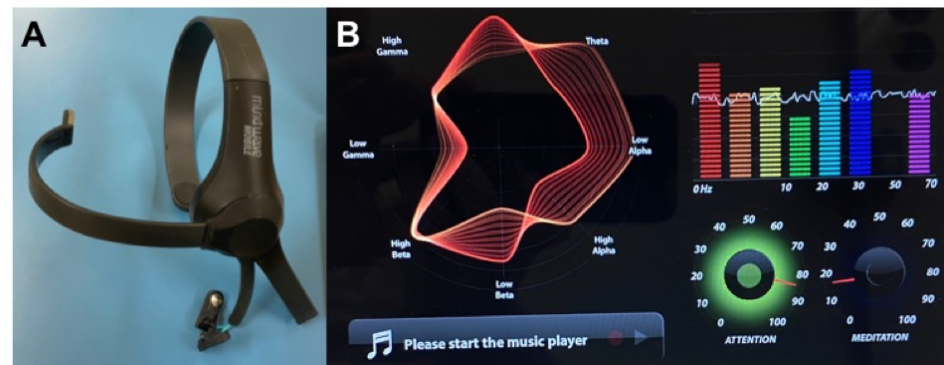
Electroencephalography device and its Neurosky driver software.

In this study, we will use TuSion software to design the gaming environment and an EEG device and its Neurosky driver software. TuSion is free software designed to help improve cognitive skills while playing (Figure 3). The game design scheme was developed considering the following points: used hardware must be cheap and easy to handle, it should be simple to get a novice to get used to it in a short amount of time, and it should use neurofeedback to give a visual representation of attention levels and thus help improve it in a more attractive way. The ThinkGear software provided with the EEG device is used to connect the EEG device to the computer. It allows special apps and games to run according to the mental states detected by the EEG earplug with NeuroSky’s ThinkGear sensor. It is provided as an executable for Mac OS X and Windows platforms. Hardware includes a laptop or smartphone and a Neurosky “Mindwave Mobile” EEG device. The hardware for acquiring brain waves consists of a sensor that touches the forehead to collect data centered on the frontal cortex, the contact, and the reference points located in the earlobe. Data is processed using the integrated chip included in it. Both the eSense Meter (meditation and attention) and raw brain waves are calculated on the ThinkGear chip. The EEG electrode is placed on the user’s forehead (on the frontal cortex) during gameplay. The earplug safely measures and produces EEG power spectra (alpha waves, beta waves, etc), attention, meditation, and blink values. Attention and meditation are indicated and reported on a meter with a relative eSense scale from 1 to 100. This scale has a set of grouping schemes for the ranges of values, and a certain mental state is assigned to it. Values between 20-40 are reduced levels and 1-20 are considered a heavily reduced eSense. A value in the range of 40 to 60 is considered neutral. Values above 60 are considered values above normal or “high.” Values in the range of 80-100 are considered high eSense levels. Attention as an unsigned 1-byte value indicates the intensity of the user’s level of mental “focus” or “attention,” as occurs during intense concentration and directed (but steady) mental activity. Its value ranges from 0 to 100 (Figure 1). Distractions, wandering thoughts, lack of focus, or anxiety decrease attention meter levels [19,20].

### Statistical Analysis

For the sociodemographic and epidemiological description of the groups, we will use the usual procedures of descriptive statistics such as calculation of frequencies and measures of central tendency and dispersion. The Shapiro-Wilk test will be performed to assess the assumption of normality of the outcome variables.

For analysis of paired and independent samples using a *t* test for comparisons within and between intervention groups or applying equivalent nonparametric tests (according to the results of the Shapiro-Wilk test) before and after the intervention, *P* values <.05 will be considered significant. Data will be organized and tabulated using SPSS (IBM Corp).

### Study Schedule

The schedule for the study is shown in Table 1.

**Table 1:** The schedule of enrollment, interventions, and assessments.

			Study period												
			Enrollment		Allocation		Postallocation								Close-out
			–*t*_1_		0		*t*_1_		*t*_2_	*t*_3_		*t*_4_		*t*_*x*_	
**Enrollment**															
	Eligibility screen	✓^a^													
	Informed consent	✓													
	Allocation			✓											
**Interventions**															
	Intervention A					✓		✓		✓	✓				
	Intervention B					✓		✓		✓	✓				
**Assessments**															
	Stroop test/US Doppler	✓		✓									✓		
	TDCS^b^ + neurofeedback							✓		✓	✓				

^a^Included at this time point.

^b^TDCS: transcranial direct current stimulation.

### Outcome Variables

The guiding theme of this study is to use tools or strategies to improve learning and promote better retention. The primary expected result is a greater correctness of answers in two phases of the Stroop test (word reading and color recognition) and a decrease in test execution time.

Secondary variables are based on physiological responses of cerebral blood flow where we expect to find an increase in velocity and systolic and diastolic peaks. This hypothesis is based on functional magnetic resonance studies where the results show an increase in local blood flow after the use of TDCS.

## Results

The trial and enrollment began in July 2022. The statistical analysis for the secondary outcomes are currently being performed.

## Discussion

We believe that the resources used in this study can help improve some aspects of declarative memory, since learning and memory processes modify the brain. Strategies to enhance the acquisition, storage, and use of information must be able to sensitize (motivate) and involve volunteers in the learning process, thus clarifying their role.

The performance of a task depends on ongoing brain activity that can be influenced by attention, excitement, or motivation. Scientific studies confirm that mindfulness leads to better performance, health, and well-being. However, these factors that modulate cognitive efficiency are nonspecific, can be difficult to control, and are not suitable to specifically facilitate neural processing.

The first stages of memory consolidation involve the stabilization of structural and functional neural changes and other neural changes generated by learning. New methods of memory consolidation optimization are being suggested (authors) in the role of neuromodulation.

The human brain is a highly interconnected network with high information processing efficiency. This efficient processing network operates through specialized structures and functions [21]. Among the essential functions of humans, memory requires significant attention from researchers because it is the ability to store and evoke learned knowledge [2]. The storage and use of learned information in the brain are fundamental for the interaction and modulation of human behavior and the adaptation and interaction of individuals with their environment [22].

In recent years, there has been an increase in technological improvement and neuroscientific discoveries aimed at new forms of training focusing on increasing memory performance [23].

As already described, neurofeedback is a brainwave training technique used to improve performance in terms of creativity, attention, and memory [24]. It has also been used as a potential cognitive and behavioral adjuvant for healthy individuals [6,25]. However, the effectiveness of neurofeedback with healthy participants received criticism from the scientific community since most studies failed to provide evidence for changes in behavioral and electrophysiological measures, mainly due to methodological deficiencies such as the lack of a sham/control group [26]. In response to this lack of reliability, Wang and Hsieh [3] researched young and older adult individuals to investigate the effectiveness of the cortical activity training protocol on attention and working memory performance. The authors observed better performance in older adults, with a significant improvement in memory among the young [3].

Although recent discoveries are addressing and answering old and new questions, the current findings provide insights into the underlying mechanisms of neurofeedback training in cognitive function. Furthermore, the results indicate that an intervention program protocol could be effective against cognitive aging and memory decline [3].

The future of neurofeedback research lies primarily in mobile recording and real-time feedback of the emotional and cognitive states of the individuals being evaluated. Due to volume conduction (the ability to measure electrical potentials at a distance from their source generators), single channels, regardless of where they are placed on the scalp, can capture a substantial fraction of all brain dynamics. Indeed, Rebolledo-Mendez et al [25] used attention levels as an input to interface systems between the brain and a computer, and found that the device provides accurate readings regarding attention since there is a positive correlation between measured and self-reported attention levels. However, setbacks observed by the authors included difficulties using the device due to the size of the head, interference with the hair, and no indicator for low batteries [25].

The primary reason for selecting this equipment is that it is a single dry electrode system, a type of EEG that does not require any substance or solution between the skin and the electrode. This feature makes the testing process more agile and reduces the individual’s discomfort. Johnstone et al [27] reported that the results obtained with this type of EEG are comparable to other devices that use different sensors and conductive substances. Additionally, it is easy to use and access the data since the equipment communicates with a computer via Bluetooth, making analyses and treatment more efficient. Furthermore, this technology comes at a low cost. These characteristics facilitate research and allow acquisition by any individual without substantial training [27].

The other device suggested for applying this protocol uses TDCS. This technique has been shown to elicit lasting effects in different protocols and diseases. TDCS provided the beneficial effect of anodic direct current stimulation of increased excitability in simple reaction times and implicit motor learning when stimulating the primary motor cortex [28] and improved learning of a visual-motor coordination task by stimulating the primary motor area or the V5 visual area [29].

Regarding its action on working memory, Fregni et al [11] demonstrated that anodic stimulation in the left DLPFC improves working memory performance. This effect depends on the polarity of the stimulation and is specific to the stimulation site. The results of Fregni et al [11] were further corroborated by Javadi and Walsh [30], who performed two experiments involving 32 participants who performed a series of word memorization tasks. This task was performed during simulated anodic and cathodic stimulation to the left DLPFC. Participants in the first experiment performed the same task with anodic TDCS of the primary motor cortex (M1). The results indicated that active stimulation of the left DLPFC leads to an improvement or attenuation of verbal memorization depending on the polarity of the stimulation. For example, during encoding, anodic stimulation of the left DLPFC improved memory, while cathodic stimulation of the same area impaired memory performance in later recognition. Anodic stimulation of M1 did not affect later recognition [30]. Smirni et al [31] observed and confirmed the same results.

To date, we have not found scientific studies using the association of the two techniques. We believe that using neurofeedback in conjunction with TDCS can potentiate the effects of training and thus provide greater effectiveness and long-term effects on the memory of individuals. In the long term, we expect that our results might further comprehensive programs for these conditions.

## Abbreviations

DLPFC: dorsolateral prefrontal cortex
EEG: electroencephalogram
TDCS: transcranial direct current stimulation

## References

[1] Ricker T, Nieuwenstein M, Bayliss D, Barrouillet P (2018). Working memory consolidation: insights from studies on attention and working memory. Ann N Y Acad Sci.

[2] Ortega-de San Luis C, Ryan TJ (2022). Understanding the physical basis of memory: molecular mechanisms of the engram. J Biol Chem.

[3] Wang J, Hsieh S (2013). Neurofeedback training improves attention and working memory performance. Clin Neurophysiol.

[4] Friehs MA, Dechant M, Vedress S, Frings C, Mandryk RL (2020). Effective gamification of the stop-signal task: two controlled laboratory experiments. JMIR Serious Games.

[5] Vourvopoulos A, Liarokapis F (2014). Evaluation of commercial brain–computer interfaces in real and virtual world environment: a pilot study. Comput Electrical Eng.

[6] Jurewicz K, Paluch K, Kublik E, Rogala J, Mikicin M, Wróbel A (2018). EEG-neurofeedback training of beta band (12-22Hz) affects alpha and beta frequencies - a controlled study of a healthy population. Neuropsychologia.

[7] Luctkar-Flude M, Groll D (2015). A systematic review of the safety and effect of neurofeedback on fatigue and cognition. Integr Cancer Ther.

[8] Jeunet C, Glize B, McGonigal A, Batail J, Micoulaud-Franchi J (2019). Using EEG-based brain computer interface and neurofeedback targeting sensorimotor rhythms to improve motor skills: theoretical background, applications and prospects. Neurophysiol Clin.

[9] Cosmo C, Baptista AF, de Sena EP (2015). Contribution of transcranial direct current stimulation on inhibitory control to assess the neurobiological aspects of attention deficit hyperactivity disorder: randomized controlled trial. JMIR Res Protoc.

[10] Buch ER, Santarnecchi E, Antal A, Born J, Celnik PA, Classen J, Gerloff C, Hallett M, Hummel FC, Nitsche MA, Pascual-Leone A, Paulus WJ, Reis J, Robertson EM, Rothwell JC, Sandrini M, Schambra HM, Wassermann EM, Ziemann U, Cohen LG (2017). Effects of tDCS on motor learning and memory formation: a consensus and critical position paper. Clin Neurophysiol.

[11] Fregni F, Boggio PS, Nitsche M, Bermpohl F, Antal A, Feredoes E, Marcolin MA, Rigonatti SP, Silva MT, Paulus W, Pascual-Leone A (2005). Anodal transcranial direct current stimulation of prefrontal cortex enhances working memory. Exp Brain Res.

[12] Boggio PS, Ferrucci R, Rigonatti SP, Covre P, Nitsche M, Pascual-Leone A, Fregni F (2006). Effects of transcranial direct current stimulation on working memory in patients with Parkinson's disease. J Neurol Sci.

[13] Moein N, Mohamadi R, Rostami R, Nitsche M, Zomorrodi R, Ostadi A, Keshtkar A (2020). Delayed auditory feedback and transcranial direct current stimulation treatment for the enhancement of speech fluency in adults who stutter: protocol for a randomized controlled trial. JMIR Res Protoc.

[14] Fregni F, Boggio PS, Nitsche MA, Rigonatti SP, Pascual-Leone A (2006). Cognitive effects of repeated sessions of transcranial direct current stimulation in patients with depression. Depress Anxiety.

[15] Nitsche MA, Cohen LG, Wassermann EM, Priori A, Lang N, Antal A, Paulus W, Hummel F, Boggio PS, Fregni F, Pascual-Leone A (2008). Transcranial direct current stimulation: state of the art 2008. Brain Stimul.

[16] Kelley NJ, Gallucci A, Riva P, Romero Lauro LJ, Schmeichel BJ (2018). Stimulating self-regulation: a review of non-invasive brain stimulation studies of goal-directed behavior. Front Behav Neurosci.

[17] Campanholo KR, Romão MA, Machado MDAR, Serrao VT, Coutinho DGC, Benute GRG, Miotto EC, de Lucia MCS (2014). Performance of an adult Brazilian sample on the Trail Making Test and Stroop Test. Dement Neuropsychol.

[18] Rogge A, Doepp F, Schreiber S, Valdueza J (2015). Transcranial color-coded duplex sonography of the middle cerebral artery: more than just the M1 segment. J Ultrasound Med.

[19] Rieiro H, Diaz-Piedra C, Morales JM, Catena A, Romero S, Roca-Gonzalez J, Fuentes LJ, Di Stasi LL (2019). Validation of electroencephalographic recordings obtained with a consumer-grade, single dry electrode, low-cost device: a comparative study. Sensors (Basel).

[20] Rogers JM, Johnstone SJ, Aminov A, Donnelly J, Wilson PH (2016). Test-retest reliability of a single-channel, wireless EEG system. Int J Psychophysiol.

[21] Langer N, von Bastian CC, Wirz H, Oberauer K, Jäncke L (2013). The effects of working memory training on functional brain network efficiency. Cortex.

[22] Schwabe L, Hermans EJ, Joëls M, Roozendaal B (2022). Mechanisms of memory under stress. Neuron.

[23] Campos da Paz VK, Garcia A, Campos da Paz Neto A, Tomaz C (2018). SMR neurofeedback training facilitates working memory performance in healthy older adults: a behavioral and EEG study. Front Behav Neurosci.

[24] Gruzelier JH (2014). EEG-neurofeedback for optimising performance. I: a review of cognitive and affective outcome in healthy participants. Neurosci Biobehav Rev.

[25] Rebolledo-Mendez G, Dunwell I, Martínez-Mirón E, Vargas-Cerdán MD, de Freitas S, Liarokapis F, García-Gaona AR, Jacko JA (2009). Assessing NeuroSky’s usability to detect attention levels in an assessment exercise. Human-Computer Interaction. New Trends 13th International Conference, HCI International 2009, San Diego, CA, USA, July 19-24, 2009, Proceedings, Part I.

[26] Rogala J, Jurewicz K, Paluch K, Kublik E, Cetnarski R, Wróbel A (2016). The do's and don'ts of neurofeedback training: a review of the controlled studies using healthy adults. Front Hum Neurosci.

[27] Johnstone SJ, Blackman R, Bruggemann JM (2012). EEG from a single-channel dry-sensor recording device. Clin EEG Neurosci.

[28] Lefaucheur J, Antal A, Ayache SS, Benninger DH, Brunelin J, Cogiamanian F, Cotelli M, De Ridder D, Ferrucci R, Langguth B, Marangolo P, Mylius V, Nitsche MA, Padberg F, Palm U, Poulet E, Priori A, Rossi S, Schecklmann M, Vanneste S, Ziemann U, Garcia-Larrea L, Paulus W (2017). Evidence-based guidelines on the therapeutic use of transcranial direct current stimulation (tDCS). Clin Neurophysiol.

[29] Antal A, Nitsche M, Kruse W, Kincses T, Hoffmann K, Paulus W (2004). Direct current stimulation over V5 enhances visuomotor coordination by improving motion perception in humans. J Cogn Neurosci.

[30] Javadi AH, Walsh V (2012). Transcranial direct current stimulation (tDCS) of the left dorsolateral prefrontal cortex modulates declarative memory. Brain Stimul.

[31] Smirni D, Turriziani P, Mangano GR, Cipolotti L, Oliveri M (2015). Modulating memory performance in healthy subjects with transcranial direct current stimulation over the right dorsolateral prefrontal cortex. PLoS One.

